# MEASUREMENT MATTERS IN EPIDEMIOLOGIC STUDIES OF SENSORY HEALTH AND AGING

**DOI:** 10.1093/geroni/igad104.0761

**Published:** 2023-12-21

**Authors:** Jennifer Deal, Bryan James, Alison Abraham

## Abstract

Valid and reliable measurement is a cornerstone of epidemiologic data collection and analysis. Sensory health is important for maintaining cognitive, physical and social function with age, but multiple measures of sensory health exist, and not all are the same. For example, audiometric hearing loss reflects the cochlea’s ability to process sound, while self-report captures an individual’s perception of their hearing ability within their social and environmental context. How we measure sensory function has important ramifications for prevalence estimates and results from association studies, and therefore, public health and clinical decision-making. Sensory loss may also have important implications for the measurement of other factors in aging research. For example, neurocognitive tests are administered aurally or on paper, which could present a challenge for older adults with sensory loss. This session, jointly sponsored by the Sensory Health and Epidemiology of Aging Special Interest Groups, focuses on the intersection of measurement and sensory health. We will report on vison and hearing measures in a large, nationally representative study of Medicare Beneficiaries, with a focus on the recent addition of objective measures of vision and hearing loss, and their implications for sensory loss prevalence estimates, particularly for the oldest old. We will also discuss how sensory loss impacts cognitive testing, including best practices for measuring cognitive function in older adults. Finally, we will present on how choice of hearing measure (self-report vs. audiometry) has important ramifications for population attributable fraction estimates, with direct implications for public health intervention for dementia prevention. This is a collaborative symposium between the Epidemiology of Aging and Sensory Health Interest Groups.

